# Illustrating potential effects of alternate control populations on real-world evidence-based statistical analyses

**DOI:** 10.1093/jamiaopen/ooab045

**Published:** 2021-06-16

**Authors:** Yidi Huang, William Yuan, Isaac S Kohane, Brett K Beaulieu-Jones

**Affiliations:** Department of Biomedical Informatics, Harvard Medical School, Countway Library, Boston, Massachusetts, USA

**Keywords:** informatics, real-world evidence, association testing, phenotyping

## Abstract

**Objective:**

Case–control study designs are commonly used in retrospective analyses of real-world evidence (RWE). Due to the increasingly wide availability of RWE, it can be difficult to determine whether findings are robust or the result of testing multiple hypotheses.

**Materials and Methods:**

We investigate the potential effects of modifying cohort definitions in a case–control association study between depression and type 2 diabetes mellitus. We used a large (>75 million individuals) de-identified administrative claims database to observe the effects of minor changes to the requirements of glucose and hemoglobin A1c tests in the control group.

**Results:**

We found that small permutations to the criteria used to define the control population result in significant shifts in both the demographic structure of the identified cohort as well as the odds ratio of association. These differences remain present when testing against age- and sex-matched controls.

**Discussion:**

Analyses of RWE need to be carefully designed to avoid issues of multiple testing. Minor changes to control cohorts can lead to significantly different results and have the potential to alter even prospective studies through selection bias.

**Conclusion:**

We believe this work offers strong support for the need for robust guidelines, best practices, and regulations around the use of observational RWE for clinical or regulatory decision-making.

**LAY SUMMARY**

Real-world evidence (RWE) refers to healthcare data generated in the course of routine clinical practice, including electronic health records and claims from health insurers. Compared to clinical trials, which often enroll curated cohorts and follow stringent protocols, RWE can capture broader and generalized patient characteristics and care practices. For this reason, there is growing interest in using RWE to evaluate the effectiveness of therapeutic interventions. Given the readily available nature of RWE, it can be difficult to evaluate the validity of results in the context of testing multiple hypothesis. Our study illustrates this vulnerability in RWE analyses by testing for association between diabetes and depression in RWE. To do this, we make small variations to the cohort definitions and find this alters the size and significance of the measured association as a result. These variations could be the result of multiple groups asking similar questions of the data, an individual asking the same question in different ways or a bad actor seeking to achieve a specific result for professional or financial motives. In light of our results, we make several recommendations to the scientific community regarding study robustness and reporting transparency.

## BACKGROUND AND SIGNIFICANCE

The FDA has shown a strong interest in the utilization of real-world evidence (RWE) to enhance or replace aspects of the regulatory process.^1^^,^^2^ Recently, the FDA increased their participation in a partnership in RWE for oncology^3^ and the pace of accelerated approvals has increased substantially from 2012.^4^^,^^5^ These actions have been met with mixed reactions,^6^ especially regarding attempts to replace traditional randomized controlled trials (RCTs) with RWE-based comparative effectiveness analyses.^7^ The important aspects of the utilization of RWE in the regulatory process we consider in this article are (1) lack of pre-registration, (2) protection against intentional or unintentional multiple testing, and (3) the potential for financial incentives to drive the strategic selection of a cohort given prior testing of retrospective data.

The digitization of medical records and administrative data have made research using RWE increasingly prevalent, and RWE has the potential to be an incredible resource to the research community.^8^^,^^9^ RWE has already enabled the study of patient-level health outcomes at an unprecedented scale, with innovative study designs that address questions such as (1) genetic heritability of different phenotypes with large scale twin studies^10^ and (2) prescribing patterns in the opioid epidemic.^11^

Due to the fact that RWE is easily available, with numerous datasets that can be purchased from commercial vendors (eg, IBM Marketscan,^12^ Optum,^13^ Premier Healthcare Database^14^) it is difficult to enforce traditional pre-registration that typically accompanies active enrollment comparative effectiveness studies (eg, RCTs). Because of this lack of pre-registration, it is more difficult and potentially impossible to determine if multiple groups have tested the same hypothesis especially given the publication bias toward positive results.^15^ When the inability to detect multiple testing is combined with the financial incentives inherent to the regulatory process for both new therapies and post-approval surveillance, there exists a possibility for bad actors to exploit the ability to intentionally multiple test or “p-hack”.^16^ Evidence to the potential for actions of this nature can be seen in recent events such as the data manipulation that occurred during the approval of Zolgensma.^17^^,^^18^ It is important to note that current RCTs are not immune to selection bias as demonstrated by the fact that they commonly include younger^19^ healthier participants.^20^ This leads to generalizability concerns for RCTs but at least in this case, due to randomization, there is no ability to pre-select the case or control groups.

## OBJECTIVE

In this work, we demonstrate the ability to profoundly affect the odds ratio (OR) for an association between depression and type 2 diabetes mellitus (T2D) by making minor alterations to the control definition using published T2D phenotyping algorithms from eMERGE.^21^ These algorithms were originally designed to overcome the challenges in identifying patient cohorts in electronic health records.^22^ We hypothesized that the requirement for a glucose test in the eMERGE may select for controls that are less healthy on average than the overall potential control population. The requirement for a glucose test can be compared to complete case analyses which can unintentionally create biases.^23^^,^^24^ In this case, this requirement may rule out a portion of the youngest, healthiest population where a physician does not believe a glucose test is necessary. If the presence of a value is not missing completely at random, its absence may be informative about the actual value.^25^ For example, the presence of a glucose test may indicate a higher prior for having an abnormal blood glucose level because a physician was concerned enough to order the lab test.

We considered a previously published association between depression and T2D and evaluate how the association changes with small permutations to the control population for T2D. Comorbidity between T2D and depression is well documented.^26–28^ The causal relationship between these diseases is best characterized as complex, and evidence exists to support both that depression elevates the risk of T2D and vice versa, as well as the hypothesis that both diseases share common etiology.^27^ This experiment demonstrates a need to show that findings derived from RWE are robust to small permutations in the included population.

Existing literature on case–control methodology has focused on confounding due to lack of randomization as well as biases in study design.^6^^,^^7^^,^^29^ Confounding due to unmeasured variables is a major concern, and makes it difficult to determine causal relationships from observational data.^25^ Other pitfalls include the nonrandom assignment of exposures^26^ and the nonrandom selection of participants.^29^ Control selection has been identified as a crucial component of case–control study design, although best-practice recommendations are centered around matching techniques to control for confounding effects.^30^^,^^31^ Multiplicity is discussed in the context of multiple hypothesis testing in genetic association studies,^32^ but we were unable to find existing work studying permutations in study design. Previous work has shown that the modeled effect size of mortality risk factors can be profoundly sensitive to model selection,^33^ suggesting to us that association results may also be sensitive to permutation in study design.

## MATERIALS AND METHODS

We performed all analyses using a large de-identified administrative claims dataset including more than 75 million individuals for the timeframe from January 1, 2008, through August 31, 2019. This database does not include any race or ethnicity data and its usage has been deemed to be de-identified non-human subjects research by the Harvard Medical School Institutional Review Board, therefore waiving the requirement for approval. The database includes member age, biological sex, and enrollment data, records of all covered diagnoses and procedures as well as medication and laboratory results for a substantial subset of the covered population. ICD-10-CM codes present in the dataset were mapped to ICD-9-CM (Supplementary Table S1) for compatibility with eMERGE phenotype definitions.

**Figure 1.**
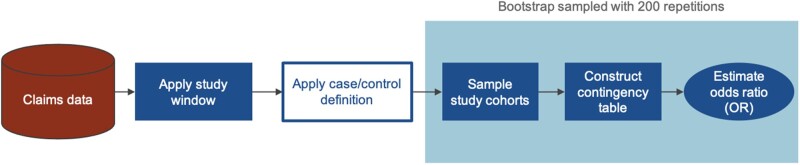
Overview of study design to evaluate the effect of alternate control groups on the association between depression and type 2 diabetes mellitus.

**Figure 2.**
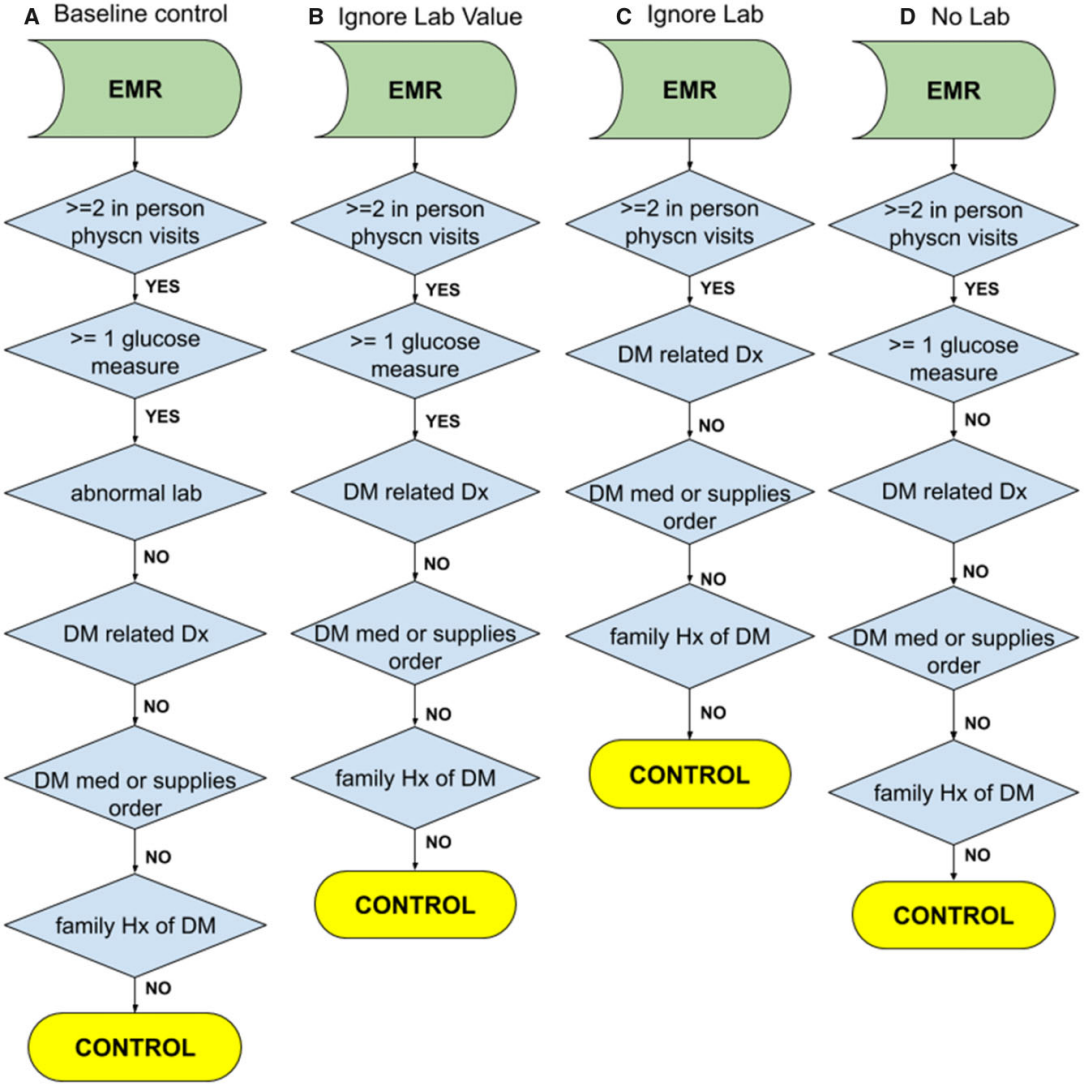
Control algorithms for type 2 diabetes mellitus. (A) Baseline controls as defined by eMERGE. (B) Controls where the glucose lab value is ignored. (C) Controls where whether the member has had a glucose lab or not is ignored. (D) Controls where the member has not had a glucose test.

An overview of the study design is shown in Figure 1. To match cohorts on coverage status, members were required to have at least 4 years of continuous enrollment to qualify, and only the first 4 years were considered when defining phenotypes. Case and control populations were generated by applying various definitions (Figure 2) to the first 4 years of claims. Case and control cohorts were sampled from populations of various sample sizes (*n* = 1000, 2000, 5000, 10 000) to test for association between depression and T2D status. Association testing was performed using the Fisher’s exact test to calculate the OR and associated *P*-value. Each test was resampled 200 times using the bootstrap method to obtain a sampling distribution for the OR. Tests were performed with and without age and sex matching and the results compared.

T2D case status was determined using an adaptation of the eMERGE T2D phenotyping algorithms^31^ for claims data. Each distinct claim was considered a separate visit for the determination of visit count. Diagnoses and medications were determined from medical and pharmacy claims, respectively. Ingredient-level RxNorm codes were mapped to NDC codes using the RxNorm API.^34^ In the absence of clinical notes or structured questionnaires, family history was determined using ICD code V18.0 in medical claims. This is a known limitation of using claims data with comprehensive medical histories.

Multiple control groups were defined based on the eMERGE T2D control algorithm with variations on the lab testing requirement (Figure 2). The eMERGE algorithm considers tests for fasting glucose (LOINC 1558-6), random glucose (2339-0, 2345-7), and hemoglobin A1c (4548-4, 17856-6, 4549-2, 17855-8) with defined thresholds for abnormal values.

We include these LOINC codes when considering lab values. Within the claims data, all lab orders (CPT codes) are available but only a subset of lab values are passed back to the private insurer. This subset is based on contracts with national laboratories to provide for the flowthrough of lab values. In total, 7 326 083 unique members have CPT codes for glucose labs with a total of 35 895 150 tests being done. Of these, 4 742 924 unique members have results available or a total of 19 175 213 tests performed. When only checking to see if a lab test is ordered, we add procedural (CPT 82947, 80047, 80048, 80053, 80069, 83036) codes to determine the presence of a test.

The groups are defined as follows:

1. eMERGE T2D control (Baseline control).
2. eMERGE T2D control still requiring lab test presence but without requirement on lab test result (Ignore lab value).
3. eMERGE T2D control without requirement on lab test presence (Ignore lab).
4. eMERGE T2D control requiring no lab test (No lab).

Exposure status for depression was defined using the Clinical Classifications Software (CCS) rollup for depressive disorders (single-level CCS diagnosis category 6572). Members with a qualifying enrollment period without a depression diagnosis were defined to be non-exposed for the purposes of this association with the acknowledgement that this does not rule out a diagnosis prior to enrollment but indicates that there is not active care being provided for depression.

All analyses were performed using queries in Microsoft^®^ SQL Server 2017 and Python™. Statistical calculations were performed using NumPy and SciPy. Visualizations were created using Matplotlib and Seaborn. All source code is available in archival form on Zenodo^35^ and on Github as a Jupyter notebook https://github.com/brettbj/association-robustness.

## RESULTS

Table 1 summarizes the demographic characteristics of the different groups. Fact count is determined as the total number of ICD codes recorded per patient per year in the qualifying 4-year window. Cases are on average older and have higher fact count than controls. Furthermore, controls who had lab testing are older still and have higher fact count than those who did not have lab testing.

**Table 1.** Demographic statistics under different control definitions

	Case population	Baseline control	Ignore lab value	Ignore lab	No lab
Members	381 412	2 868 491	6 204 015	10 286 072	4 054 244
% Male	51.90	42.90	45.09	47.84	52.10
% Female	48.10	57.10	54.91	52.16	47.89
Age	64.16 (12.47)	48.70 (17.04)	50.74 (19.19)	43.12 (21.99)	31.29 (20.69)
% w/depression	17.51	14.24	14.94	10.70	4.22
Total facts per year	53.54 (55.10)	25.91 (29.86)	29.07 (34.29)	21.99 (29.54)	11.12 (14.64)

**Figure 3.**
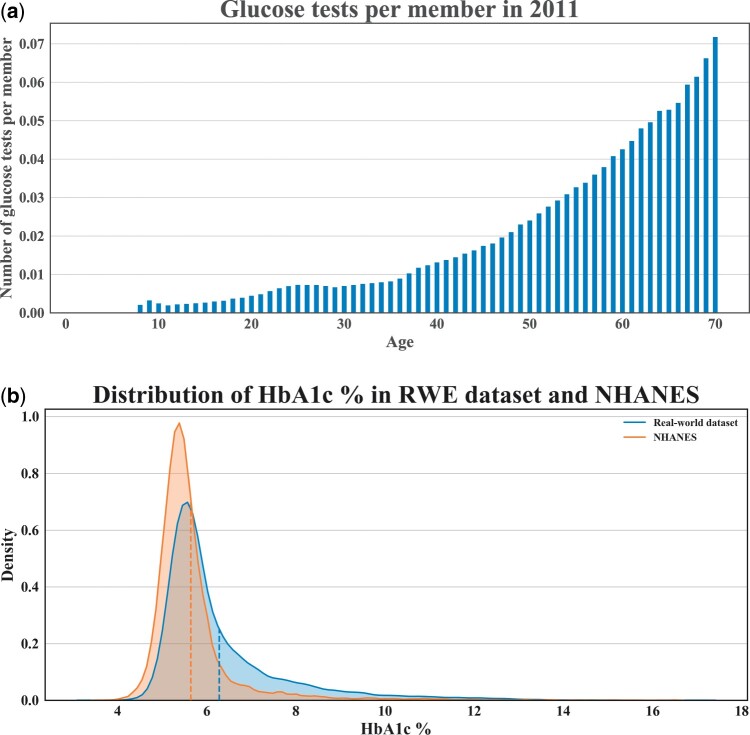
Potential selection effects from glucose lab testing requirement. (A) Number of glucose tests per member during 2011 by age. (B) Distributions of HbA1c value from our dataset compared with privately insured individuals from the National Health and Nutrition Examination Survey (NHANES).

Figure 3A shows that members receive more glucose testing as they age (per member per year). This indicates that requiring a glucose test may cause the control population to be older on average. Indeed, the control population with glucose testing is on average 16 years older than the control population without. Figure 3B shows that physician-ordered HbA1c lab values in our dataset tend to be higher (mean = 6.28, median = 5.8) than in an age- and gender-matched cohort from a representative population sample in National Health and Nutrition Examination Survey (mean = 5.64, median = 5.4). Taken altogether, these indicate that requiring glucose testing as part of the eMERGE control algorithm may select for a population closer to the case population than the entire potential control population.

**Figure 4.**
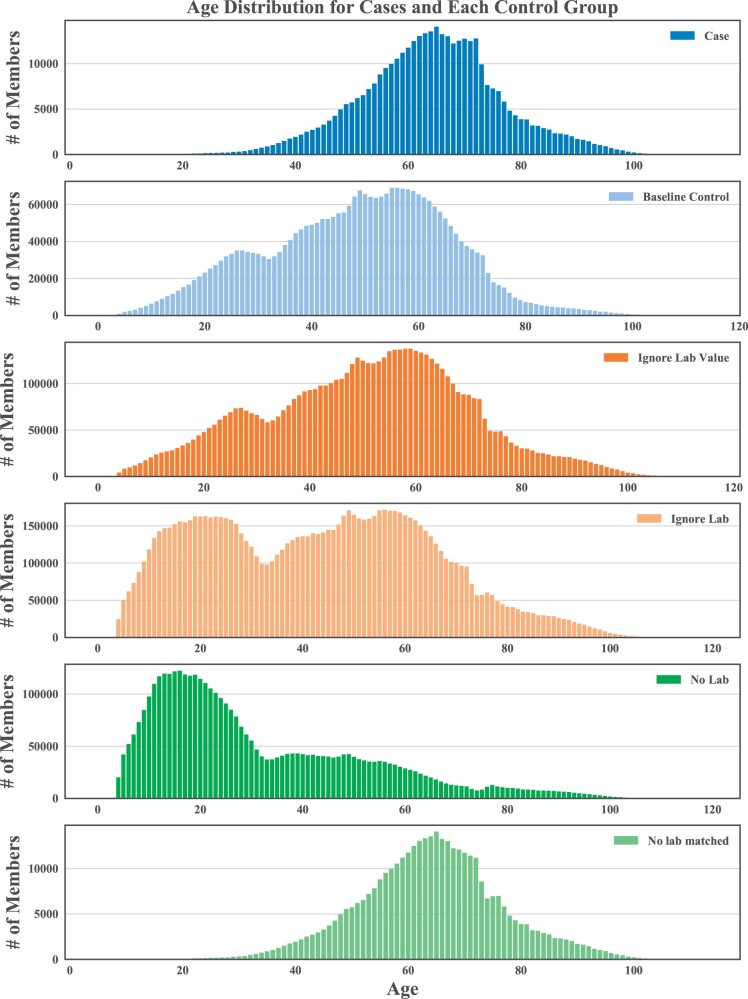
Age distributions under each different control definition.

Adjusting the glucose lab requirement in the control definition results in different age distributions (Figure 4). Matching on age and sex corrects for these differences but the percentage of cases with depression diagnoses and the total number of diagnoses, or facts, remains higher in the case population (Table 2 and Supplementary Tables S2–S4).

**Table 2.** Case and control summary statistics after matching

	Case population	No labs matched
Members	374 719	374 719
% Male	51.04	51.04
% Female	48.96	48.96
Age	64.00 (12.54)	64.00 (12.54)
% members with depression	17.61	6.61
Total facts per year	53.25 (55.11)	13.50 (18.85)

We found a significant association between type 2 diabetes and depression in most bootstrapped samples. When sampling case/control cohorts at *n* = 10 000, only 2 out of 1600 total randomizations resulted in a non-significant test. Within the matched populations, requiring that controls have a glucose test ordered but ignoring its value (median OR, 1.168, 95% confidence interval [CI], 1.085–1.259) results in a slightly weaker association compared to the baseline control (median OR, 1.277, 95% CI, 1.183–1.378). This reinforces the existence of a true association, as this variation may be inducing some case contamination in the control group. Testing against the no lab control group (median OR, 2.655, 95% CI, 2.417–2.918) yields a much higher OR compared to baseline. Testing against the ignore lab control group (median OR, 1.309, 95% CI, 1.213–1.413) results in an OR between the baseline control and the no lab control group.

**Figure 5.**
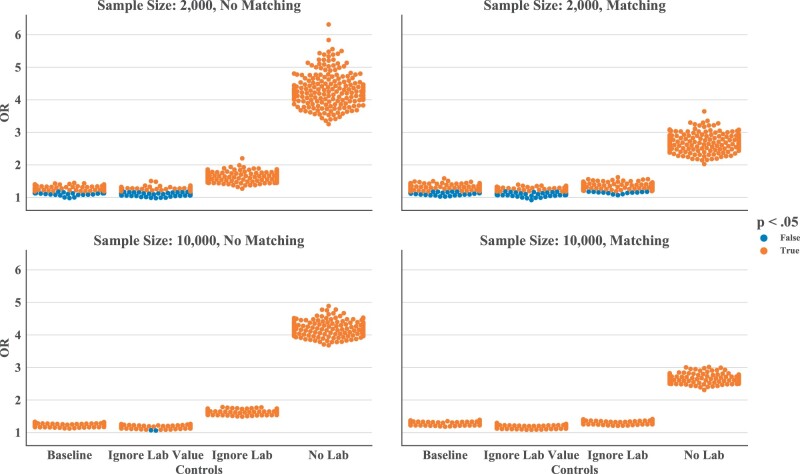
Odds ratio among each of the different control groups for 200 samples. (A) Sample size of 2000 without matching, (B) sample size of 2000 with matching on age and sex, (C) sample size of 10 000 without matching, and (D) sample size of 10 000 with matching on age and sex.

At lower sample sizes (*n* = 2000), the ORs estimates are more widely distributed (95% CI range is 2.26 times greater for the baseline group, 2.26 times greater for the ignore lab value group, 2.25 times greater for the ignore lab group, and 2.25 times greater for the no lab group). In the no lab control setting, reducing cohort size does not affect the significance of the test, given the margin between the OR and one. When the OR is closer to one, smaller cohort size and the less precise estimate may lead to a change in direction for the OR and tests may lose significance. As many as 111/200 tests in the ignore lab value setting in Figure 5B tested non-significant.

## DISCUSSION

We applied retrospective case–control study design in a large administrative claims dataset to test for association between depression and T2D using multiple control group definitions. Taken altogether, the evidence suggests a true association exists between depression and T2D, but we do not yet attempt to determine directionality or causality. We found that permutations to the control definition which we believe to be reasonable led to changes of results. This was shown through shifts in the demographic structure of the control population, as well as differences in the OR of association. With age and sex matching, these differences are tempered, but still significant. Increasing the sample size reduces the variance between replicates, but the OR shift following changes in control definition remains.

These results indicate that it would be possible for bad actors to manipulate results based on RWE in a manner that is difficult to detect without knowing all experimental parameters used by the bad actors. It is therefore critical to establish best practices regarding transparency, neutrality, conflicts of interest, data provenance, pre-registration, cohort selection, sample sizes, and reporting of results prior to using RWE as a major component of the regulatory process.

There were several limitations to this study. While we measure an association between depression and T2D, our study does not attempt to conclude whether depression elevates the risk of developing T2D or vice versa. Furthermore, it is unclear what biases are being encoded by the different definitions and how they drive changes in OR. Moreover, we made several approximations in repurposing the eMERGE T2D algorithm for claims data which may have affected its specificity. No one definition of a control definition is likely to be robust for all purposes and each of the proposed modifications has potential weaknesses. In addition to the lack of formal determination of family history, systematic biases may exist in the availability of lab test values. Finally, the use of a private insurance dataset selects against older segments of the population who may qualify for Medicare, as well as unemployed/lower socio-economic status segments who cannot afford private insurance.

## CONCLUSION

This study demonstrates that effect size in retrospective association studies may be maximized by cherry-picking a control group. By using reasonable alternate definitions of the control phenotype in tests of association between T2D and depression, we are able to meaningfully change the makeup of the comparison group, leading to significant differences in the OR of association. We suggest that the ability to strategically select a control group is not limited to association studies but extends to most RWE and retrospective studies. To mitigate the risk of publishing an unsound result, we recommend: (1) RWE-based studies use and do not modify externally generated and validated pre-defined eligibility criteria, (2) if not possible RWE-based studies should pre-register eligibility criteria and protocol prior to obtaining data, (3) report all permutations tested with results for each permutation (potentially through the use of an independent audit system), (4) avoid subsampling or report all subsamples with a variety of random seeds, and (5) report a Bayes estimate of the likelihood that the study will replicate.^36^ Several of our suggestions (eg, 1–4) do not remove the potential of a bad faith actor and rely on scientific integrity. Given this fact, when these analyses are used as evidence for decisions with a real-world, patient safety or financial impact, study designs should be validated, and results replicated by an independent neutral body.

## FUNDING

This work was supported by the National Library of Medicine grant number (T15LM007092), the National Institute of Neurological Disorders and Stroke grant number (K99NS114850), and the National Institutes of Health BD2K program.

## AUTHOR CONTRIBUTIONS

BKB-J, YH, and ISK designed the study. Quantitative analysis was performed by YH and BKB-J. All authors contributed to the study design and results interpretation. YH and BKB-J were responsible for the initial draft of the manuscript. All authors reviewed, edited, and approved the final manuscript.

## SUPPLEMENTARY MATERIAL

Supplementary material is available at *Journal of the American Medical Informatics Association* online.

## CONFLICT OF INTEREST STATEMENT

None declared.

## DATA AVAILABILITY

The dataset was made available to the Harvard Medical School Department of Biomedical Informatics as part of the Healthcare Data Science Program. The data may be available through commercial agreement with the nationwide US health insurance plan. Summary data are available from the authors upon reasonable request and with permission from the insurer. Code for analysis, generation of figures, and figure files is available at https://github.com/brettbj/association-robustness.

## Supplementary Material

ooab045_Supplementary_Data
