# Supplementary material for: Illustrating potential effects of alternate control populations on real-world evidence-based statistical analyses
Source: JAMIA Open. 2021 Jun 16;4(2):ooab045. doi: 10.1093/jamiaopen/ooab045 (PMC8206406; doi:10.1093/jamiaopen/ooab045)
Supplement: ooab045_Supplementary_Data [file ooab045_supplementary_data.docx]

# Supplemental Materials

**Supplemental Table 1:** Mapping from ICD-9 to ICD-10

*_ - indicates a single character wildcard*

*% - indicates a zero-or-more character wildcard*

| **Diagnosis** | **ICD-9-CM** | **ICD-10-CM** |
| --- | --- | --- |
| Type 1 DM | 250._1, 250._3, | E10.% |
| Type 2 DM | 250._0, 250._2, | E11.% |
| Abnormal blood glucose | 790.2% | R73.% |
| Abnormal glucose during pregnancy | 648.8% | O99.81% |
| Gestational diabetes | 648.0% | O24% |
| Glycosuria | 791.5 | R81 |
| Dysmetabolic syndrome X | 277.7 | E88.81 |
| Family history of DM | V18.0 | Z83.3 |
| Screening for DM | V77.1 | Z13.1 |
|  |  |  |
| Mood disorder other dis. | 293.83 | F06.3% |
| Major depression single episode | 296.2% | F32% |
| Major depression recurrent episode | 296.3% | F33% |
| Dysthymic disorder | 300.4 | F34.1 |
| Depressive disorder NEC | 311 | F32.9 |

**Supplemental Table 2:** Matched populations for baseline control definition

|  | Case Matched | Baseline Control Matched |
| --- | --- | --- |
| Members | 381,401 | 381,401 |
| % Male | 51.90 | 51.90 |
| % Female | 48.10 | 48.10 |
| Age | 64.00 (12.47) | 64.00 (12.47) |
| % Members with Depression | 17.51 | 13.75 |
| Total Facts Per Year | 53.50 (55.10) | 31.00 (31.90) |

**Supplemental Table 3:** Matched populations for ignore lab value control definition

|  | Case Matched | Ignore Lab Value Matched |
| --- | --- | --- |
| Members | 381,403 | 381,403 |
| % Male | 51.90 | 51.90 |
| % Female | 48.10 | 48.10 |
| Age | 64.00 (12.47) | 64.00 (12.47) |
| % Members with Depression | 17.51 | 15.00 |
| Total Facts Per Year | 53.50 (55.10) | 34.00 (36.18) |

**Supplemental Table 4:** Matched populations for ignore lab control definition

|  | Case Matched | Ignore Lab Matched |
| --- | --- | --- |
| Members | 381,403 | 381,403 |
| % Male | 51.90 | 51.90 |
| % Female | 48.10 | 48.10 |
| Age | 64.00 (12.47) | 64.00 (12.47) |
| % Members with Depression | 17.51 | 13.37 |
| Total Facts Per Year | 53.50 (55.10) | 30.00 (33.83) |
